# Structure modeling and quantitative X-ray diffraction of C-(A)-S-H

**DOI:** 10.1107/S1600576721012668

**Published:** 2022-02-01

**Authors:** Karsten Mesecke, Laurence N. Warr, Winfried Malorny

**Affiliations:** a Hochschule Wismar, Philipp-Müller-Straße 14, 23966 Wismar, Germany; b University of Greifswald, Friedrich-Ludwig-Jahn-Straße 17A, 17489 Greifswald, Germany

**Keywords:** *TOPAS*, supercell approach, calcium silicate hydrates, C-(A)-S-H

## Abstract

Quantitative X-ray diffraction of nanocrystalline calcium silicate hydrate (C-S-H) and its aluminium-substituted variants (C-A-S-H) has so far been limited by a lack of appropriate structure models. In this study, atomistic structure models derived from tobermorite were combined with a supercell approach using *TOPAS*. By accounting for nanostructural features such as isolated layers, turbostratic disorder and, for the first time, fibrils, characteristic reflections and asymmetric bands were more accurately simulated than before, providing the means for phase quantification and refinement of structural sites. This improved methodology is applied to autoclaved aerated concrete and the experimental study of related hydrothermal reactions. Scanning electron microscopy indicated a fibrillar morphology for intermediate C-(A)-S-H, and energy-dispersive X-ray spectroscopy constrained its Ca/Si ratio to 1.31–1.35. As a first step, the direct quantification of C-(A)-S-H via structure models was assessed by a series of X-ray diffraction measurements using corundum as an internal standard. Secondly, the verified structure model was applied to evaluate *in situ* X-ray diffraction experiments at 457, 466 and 473 K (1.1, 1.35 and 1.55 MPa, respectively). Finally, a quantitative study of industrially produced autoclaved aerated concrete was conducted, determining 20–30 wt% C-(A)-S-H at Ca/Si ratios < 1.0. In general, the developed structure models advance the study of Portland cement concrete and related materials, including autoclaved aerated concrete, and the supercell approach may be universally applicable to other nanocrystalline materials.

## Abstract 2

Atomistic structure models of nanocrystalline calcium silicate hydrate [C-(A)-S-H] that account for nanostructural features such as isolated layers, turbostratic disorder and fibrils are obtained via a supercell approach using *TOPAS*. Quantitative X-ray diffraction of C-(A)-S-H is demonstrated for autoclaved aerated concrete and intermediates.

## 1. Introduction

Nanocrystalline calcium silicate hydrate (C-S-H) and its aluminium-substituted variants (C-A-S-H) have been intensively studied owing to their importance as the main binding phase in Portland cement concrete. They display a wide range of chemical compositions, structures and crystallinities, usually categorized in terms of their Ca/Si ratio (Richardson, 2014 ▸). C-(A)-S-H as found in Portland cement concrete marks the upper limit at Ca/Si ≃ 1.8 and shows moderate aluminium substitution at Al/Si ratios of 0.05 (Richardson *et al.*, 2010 ▸). Water contents are often described in terms of H_2_O/Si ratios, which show considerable variation between analysis methods since they also account, for example, for hydroxyl ions or adsorbed water (Lothenbach & Nonat, 2015 ▸; Roosz *et al.*, 2016 ▸). The morphologies as observed by transmission electron microscopy (TEM) range from lamellar to fibrillar and are influenced by Ca/Si ratio and chemical environment (Richardson, 2004 ▸; Richardson *et al.*, 2010 ▸; Tajuelo Rodriguez *et al.*, 2015 ▸). C-(A)-S-H is termed nanocrystalline in respect to its diffuse X-ray diffraction (XRD) patterns, which share some characteristics with those of crystalline calcium silicate hydrates, especially the layered mineral tobermorite (Nonat, 2004 ▸). Asymmetric *hk* bands can be attributed to isolated layers or, in the presence of a 001 reflection, to turbostratic disorder, which is the random translation and rotation of layers parallel to each other (Grangeon, Claret, Lerouge *et al.*, 2013 ▸; Grangeon, Claret, Linard & Chiaberge, 2013 ▸; Grangeon *et al.*, 2016 ▸, 2017 ▸). The most diffuse XRD patterns can be attributed to minute crystallite sizes along the *a* and *c* dimensions, which would be equivalent to fibrils (Richardson, 2014 ▸). Despite common terminology, C-(A)-S-H phases are not completely X-ray amorphous, nor do they contain amorphous and crystalline domains as suggested by the term semi-crystalline.

Tobermorite [Ca_4.5_Si_6_O_14_(OH)_3_·2H_2_O] is the main binding phase in autoclaved aerated concrete (AAC) and other hydrothermally cured building materials (Gundlach, 1973 ▸; Hamid, 1981 ▸). C-(A)-S-H, as the main intermediate phase in the production process, either originates from the hydration of Portland cement or forms under hydrothermal conditions from portlandite and silicate, *e.g.* quartz (Schober, 2020 ▸). The hydrothermal formation of tobermorite has been studied by both *ex situ* and *in situ* methods at temperatures of 453 K (Mitsuda, 1982 ▸), 463 K (Bernstein, 2011 ▸; Kikuma *et al.*, 2011 ▸; Matsui *et al.*, 2011 ▸; Schober, 2005 ▸) and 463–583 K (Shaw *et al.*, 2000 ▸). For example, the *ex situ* study of Schober (2005 ▸) provided phase quantifications including C-(A)-S-H for 30 or 60 min intervals. Quantifications used an internal standard and assumed a constant Ca/Si ratio for the C-(A)-S-H phase (Schober, 2020 ▸). The more recent *in situ* XRD studies of Matsui *et al.* (2011 ▸) and Kikuma *et al.* (2011 ▸) presented improved data with measurement intervals of 4.15 min. However, their evaluation of phases relied entirely on normalized integrated intensities.

Direct quantification of C-(A)-S-H via Rietveld refinement would improve the significance of *in situ* XRD studies and advance the quality control of AAC products, where larger amounts of C-(A)-S-H are known to affect key parameters such as drying shrinkage (Gundlach, 1973 ▸). Owing to the lack of a defined crystal structure, quantitative XRD has, however, proven difficult and relies on the successful application of internal or external standard methods. For instance, Garbev *et al.* (2008 ▸) simulated the C-S-H XRD pattern by whole powder pattern decomposition assuming lattice constants similar to those of tobermorite, but the actual quantification relied on the addition of corundum as an internal standard. In contrast, the external standard methods of Snellings *et al.* (2014 ▸) and Maddalena *et al.* (2019 ▸) used pseudo-Voigt profiles for quantitative comparison of peak areas relative to a reference sample. Furthermore, Jansen *et al.* (2011 ▸) examined alite hydration by the G-factor method, which was combined with an *hkl* phase model for C-S-H by Bergold *et al.* (2013 ▸) and used for the study of AAC paste hydration by Schreiner *et al.* (2018 ▸).

The importance of Portland cement concrete has led to the proposal of various structure models for C-(A)-S-H, many of them derived from tobermorite or clinotobermorite. The Ca/Si ratios were usually adjusted by the omission of bridging Si tetrahedra and increased occupancy of interlayer Ca. For example, Geng *et al.* (2017 ▸) applied anisotropic crystallite size and strain broadening to 14 Å tobermorite, whereas Renaudin *et al.* (2009 ▸) and Battocchio *et al.* (2012 ▸) used 11 Å tobermorite; the latter also constrained certain geometries (*e.g.* tetrahedral geometry for silicate) and refined atom positions without loss of coordination or inappropriate modification of bond lengths. Attempts towards a more realistic description of C-(A)-S-H including the nanostructure led to large-scale atomistic structure models composed of building blocks that cover different chemical compositions or variable configurations, or even represent surfaces (Kunhi Mohamed *et al.*, 2018 ▸). Furthermore, Richardson (2014 ▸) derived multiple hypothetical C-S-H structure models by chemical and geometrical reasoning.

The most elegant simulation of asymmetric *hk* bands, however, did not require any anisotropic crystallite size or strain broadening and was obtained from 11 Å tobermorite solely by considering turbostratic disorder (Grangeon, Claret, Lerouge *et al.*, 2013 ▸; Grangeon, Claret, Linard & Chiaberge, 2013 ▸; Grangeon *et al.*, 2016 ▸, 2017 ▸; Drits & Tchoubar, 1990 ▸). The utilized mathematical formalism may not be applicable for phase quantification, but other turbostratically disordered phases such as smectite and nontronite have been described by Rietveld-compatible supercell approaches (Ufer *et al.*, 2004 ▸; Wang *et al.*, 2012 ▸). Therefore, the objective of this study is to develop and test a similar supercell approach for C-(A)-S-H phases using *TOPAS* (Coelho, 2018 ▸) and to assess its applicability in refining *ex situ* and *in situ* XRD measurements related to the hydrothermal curing of AAC.

## 2. Materials and methods

### 2.1. Sample preparation

An AAC mixture was prepared similarly to industrial recipes from 55.6 wt% quartz sand (99.1 wt% SiO_2_; D50 23 µm), 22.2 wt% ordinary Portland cement (CEM I 52.5 R), 22.2 wt% lime (93 wt% CaO), 0.1 wt% aluminium powder and water with a water-to-solid ratio of 0.72. A specimen Ca/Si ratio of 0.63 was calculated from the chemical composition of the raw materials [evaluated by X-ray fluorescence (XRF)]. Compared with industrial recipes, a lower SO_3_ content of 0.56 wt% was obtained by partially replacing cement with lime and refraining from calcium sulfate addition. The mixture was homogenized using a dissolver blade stirrer for 3 min at 3000 min^−1^ and cast into a polytetrafluoroethylene (PTFE) mold. Foaming, subsequent setting and accelerated cement hydration took place at 340 K for 6 h. In order to produce C-(A)-S-H without much tobermorite, the mixture was briefly cured in saturated steam; heating from 373 K to *ca* 458 K (1.1 MPa) took 200 min, and the temperature of 458 K was maintained for 90 min. After cooling below 340 K and before opening, the autoclave was flushed with nitrogen to minimize carbonatization. Further drying took place at <310 K for 4 h.

Experimental mixtures for *in situ* XRD were prepared likewise from 52.8 wt% quartz sand (99.1 wt% SiO_2_; D50 23 mm), 21.0 wt% ordinary Portland cement (CEM I 52.5 R), 21.0 wt% lime (93 wt% CaO), 5.1 wt% calcium fluoride (>99 wt%; sintered at 970 K for 3 h), 0.1 wt% aluminium powder and water with a water-to-solid ratio of 0.7. In contrast to industrial recipes, calcium fluoride was added to serve as an internal standard as it was assumed to be inert. A specimen Ca/Si ratio of 0.69 was obtained, whereas the calculation without calcium fluoride would obtain Ca/Si of 0.63. The mixtures were homogenized and cast into sample trays, usually three at a time, and covered with a PTFE thread-seal tape, polyethylene (PE) foil and a planar weight. The foaming yielded an even surface of *ca* 40 × 20 mm. The samples were kept at 340 K for the first 6 h and then at room temperature for at least 36 h prior to *in situ* measurement.

### 2.2. X-ray fluorescence

Dried powder samples were mixed with lithium tetraborate in a ratio of 1:8 and fused into glass beads. The loss on ignition was determined in parallel at 1243 K for 1 h. The chemical composition was determined using a Bruker S8 Tiger wavelength dispersive X-ray fluorescence spectrometer calibrated against 27 glass bead standards.

### 2.3. Scanning electron microscopy

For scanning electron microscopy (SEM) analysis, broken-off pieces (<10 mm) were fixed to sample stubs using a conductive carbon tab; no surface coating or polishing was applied. Secondary electron images at a magnification of 10 000 were obtained with an acceleration voltage of 5 kV using an FEI QUANTA 250 field emission gun in high-vacuum mode. Quantitative energy-dispersive X-ray spectroscopy (EDX) was carried out on a Zeiss EVO MA 10 with an acceleration voltage of 15 kV and a beam current of 1.5 nA. Spectra were recorded at imprints of larger quartz grains at a magnification of 5000, with an acquisition time of 100 s. Those quartz grains are usually surrounded by homogeneous C-(A)-S-H or tobermorite which are exposed if grains are broken off. Uncertainties remain about the sample topography, which may cause inaccuracies due to microabsorption. The X-ray absorption of lighter elements increases disproportionately at longer absorption path lengths, *e.g.* underestimating oxygen and exaggerating calcium (Newbury & Ritchie, 2013 ▸). Therefore the theoretical 66.7 at.% oxygen present in mixtures of Ca(OH)_2_ and SiO_2_ was used as a selection criterion (Fig. S1).

### 2.4. X-ray diffraction

X-ray diffraction was carried out on a Bruker D8 operated in Bragg–Brentano geometry. An X-ray source (Cu 40 kV, 40 mA) with a long fine focus and a linear position-sensitive detector (LynxEye) with an opening of 2.947° 2θ were mounted at radii of 280 mm. The anti-scattering slit was set to 2.56° and an Ni *K*β filter was employed. Conventional powder samples with a diameter of 25 mm were front loaded and measured under rotation by continuous scans from 5 to 70° 2θ with a step size of 0.01°, a measurement time per step of 0.5 or 0.95 s, and Soller slits of 2.5° on the primary and secondary paths. The variable divergence slit was set to illuminate either 13 or 22 mm on the sample. Atomic displacement parameters were investigated by measurements up to 135° 2θ with a reduced sample illumination of 5 mm.

*In situ* experiments were carried out on the same instrument equipped with an autoclave chamber (Mesecke *et al.*, 2020 ▸). Each measurement was recorded within 14 min (or initially 11 min) by continuous scans from 14.2 to 50.8° 2θ (or 8.6 to 37.1° 2θ) with a step size of 0.01°, a measurement time per step of 0.2 s, a fixed divergence slit of 0.9° and a Soller slit of 2.5° on the secondary path. Sample trays were unwrapped and inserted into the chamber. Unirradiated parts were covered with a specifically shaped PTFE foil in order to prevent condensate leaching later on. After the front face screw-on lid was sealed, the still unpressurized system was pre-heated using a circulation thermostat. At 363–373 K the chamber was flushed with steam from an external boiler to ensure saturated water vapor, after which the whole system was sealed (0 min). The temperature increased steadily at a rate of 0.51–0.54 K min^−1^ until constant conditions were approached (*ca* 200 min). The studied temperatures (457, 466 and 473 K) were maintained at ±1 K for at least 6 h. Afterwards, the chamber cooled at a rate of *ca* 1 K min^−1^ and at <330 K the chamber was opened.

### 2.5. Rietveld refinement

Crystallographic information for Rietveld refinement using *TOPAS 5.0* was retrieved from the Crystallography Open Database (COD; Gražulis *et al.*, 2009 ▸) and the American Mineralogist Crystal Structure Database (AMCSD; Downs & Hall-Wallace, 2003 ▸). Custom macros were made accessible to GUI mode by modification of the *topas.inc* macro library. The following structure models were used without further modifications: anhydrite (Cheng & Zussman, 1963 ▸; COD 5000040), bassanite (Bezou *et al.*, 1995 ▸; COD 9012208), calcite (Maslen *et al.*, 1993 ▸; COD 2100992), corundum (Lutterotti & Scardi, 1990 ▸; COD 1000032), gypsum (Henry *et al.*, 2009 ▸; COD 2300258), katoite (Kyono & Arora, 2019 ▸; COD 1552362), larnite (Tsurumi *et al.*, 1994 ▸; COD 9012789) and vaterite (Demichelis *et al.*, 2012 ▸; AMCSD 0019870). Portlandite (Desgranges *et al.*, 1993 ▸; COD 1001768) was described using two structures to account for small and large crystallite sizes. For quartz (Antao *et al.*, 2008 ▸; COD 9013321), a slight correction of preferred orientation along 011 was implemented. For tobermorite (Hamid, 1981 ▸; COD 8103550) a slight correction of preferred orientation along 110 was likewise implemented and combined with an anisotropic peak broadening for triaxial cylinders at fixed parameters (*rx* 40 nm, *ry* 400 nm and *rz* 4 nm) (Ectors *et al.*, 2015 ▸; Schreiner *et al.*, 2019 ▸). Ellestadite-(OH) (Pajares *et al.*, 2002 ▸; COD 9012720) was obtained by replacing F^−^ sites with O^2−^ sites. For orthoclase (Phillips & Ribbe, 1973 ▸; COD 9000304) and phlogopite (Redhammer & Roth, 2002 ▸; COD 9002829) preferred orientation was implemented along 002 and 112, respectively. For fluorite (Batchelder & Simmons, 1964 ▸; COD 5000222) atomic displacement parameters were refined. The PTFE reflection at 18.8° 2θ observed by *in situ* XRD was fitted by a pseudo-Voigt peak profile.

All measurements were refined sequentially. *In situ* measurements severely affected by condensate droplets on the beryllium window were excluded. Fifth-order Chebyshev polynomials were used to refine the background. The sample displacement parameter had an overall margin of −0.5 to 0.0 mm and changed in relation to temperature by *ca* 0.1 mm during particular *in situ* experiments.

### 2.6. Reaction kinetics

Because there were occasional disturbances from steam and condensate, at least three *in situ* experiments were combined for each investigated temperature. In order to improve the accessibility of time-resolved phase quantities, the reaction kinetics were modeled by regression analysis using the least-squares method. The equations are empirical and usually assume a first-order reaction behavior. The parameters include the initial quantity *w*_0_, the rate constant *k*, the time of the onset of reaction *t*_0_ and the theoretical quantity after prolonged reaction *w*_ 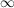 _.

## 3. Structure models for C-(A)-S-H

### 3.1. Chemical composition

On the basis of, for example, ^29^Si magic-angle spinning NMR studies, the chemistry of C-(A)-S-H can be summarized as follows. The tobermorite-like main layer comprises opposing sevenfold-coordinated Ca–O polyhedra encompassed by paired silicate tetrahedra with the formula [Ca_2_Si_2_O_7_]^2−^ (Gartner *et al.*, 2017 ▸). At low Ca/Si ratios, bridging silicate tetrahedra connect those pairs and form long chains where the negative charge is primarily balanced by protons (Table 1 ▸). With increasing Ca/Si, fewer bridging silicate tetrahedra are present and larger proportions of the charge are balanced by Ca^2+^ ions (Gartner *et al.*, 2017 ▸). The positions of the charge-balancing Ca^2+^ ions and their ligands remain uncertain. For example, in tobermorite (Hamid, 1981 ▸) there is no apparent geometry, but Richardson (2014 ▸) proposed an octahedral geometry similar to that of portlandite. In that structure, both sides of the sixfold-coordinated Ca–O octahedra share one O atom with a silicate tetrahedron and two O atoms with the main-layer Ca–O polyhedra. In isolated layers, one side is instead coordinated by water or hydroxyl groups (Gartner *et al.*, 2017 ▸). At Ca/Si ratios of 1.0, half of the bridging tetrahedra are occupied and the negative charge of the main layer is balanced by identical proportions of Ca^2+^ ions and protons. At Ca/Si = 1.3, the bridging silicate occupancy decreases further and protons are replaced by Ca^2+^ ions completely (Richardson, 2014 ▸). Assuming an occupancy of 20%, a neutral charge without protons or hydroxyl groups is maintained at Ca/Si = 1.364 (Table 1 ▸). Even higher Ca/Si ratios of up to ∼1.8 occur in saturated Ca(OH)_2_ solution, such as freshly hydrated Portland cement concrete. Because of the high alkalinity, an increasing fraction of divalent Ca^2+^ ions are replaced by two monovalent CaOH^+^ ions (Table 1 ▸) (Gartner *et al.*, 2017 ▸). For example, at Ca/Si = 1.7 the expected ratio of CaOH^+^ to the total amount of Ca is 0.4, whereas a ratio of 0.46 was determined experimentally (Thomas *et al.*, 2003 ▸). For hydrothermal solutions, the Ca(OH)_2_ saturation concentration at 453 K is one order of magnitude lower than that at room temperature (Peppler & Wells, 1954 ▸) and its alkalinity is slightly reduced (Bates *et al.*, 1956 ▸).

Aluminium replaces silicon predominantly at bridging sites. At moderate alkalinity its tetrahedral geometry is maintained, whereas at high alkalinity the availability of hydroxyl ligands induces an octahedral coordination (Kunhi Mohamed *et al.*, 2020 ▸). The additional negative charge due to Al^3+^ instead of Si^4+^ requires additional charge-balancing ions, which are Ca^2+^ or potentially alkali ions (Richardson, 2014 ▸). Since aluminium and silicon are similar in terms of their X-ray scattering ability, the addition of structural sites for Al^3+^ seems unnecessary at Al/Si 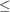 0.05.

Since structural sites for coordinating water and hydroxyl groups are incorporated into the structure models, H_2_O/Si ratios at high Ca/Si account for mild drying conditions (Lothenbach & Nonat, 2015 ▸; Richardson, 2014 ▸; Roosz *et al.*, 2016 ▸). On the other hand, the positions of adsorbed water predominant at low Ca/Si are more difficult to assume and respective H_2_O/Si ratios account for more severe drying conditions (Richardson, 2014 ▸).

### 3.2. The supercell approach

**Figure 1:**
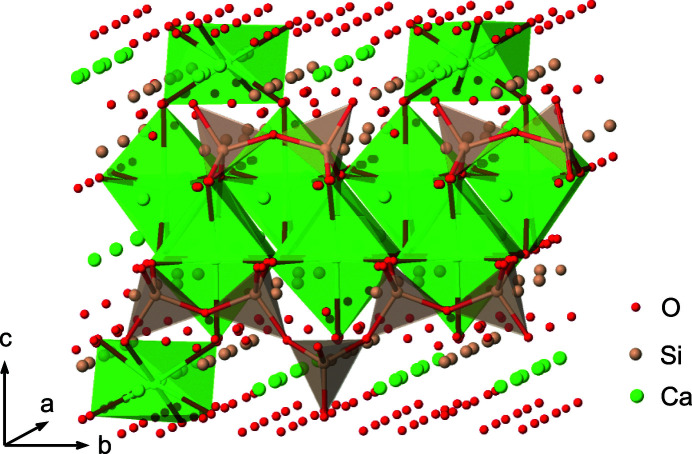
C-(A)-S-H derived from tobermorite with statistically distributed sites and exemplary marked coordination spheres. The central main-layer Ca–O polyhedra (green) and paired silicate tetrahedra (gray) are encompassed by bridging silicate tetrahedra and charge-balancing Ca–O octahedra. Created with the IUCr *Jmol* enhanced figure toolkit (McMahon & Hanson, 2008 ▸).

Nanostructures like isolated layers, turbostratically disordered layers and fibrils can be simulated by a supercell approach using *TOPAS*. Structural sites in *TOPAS*-compatible syntax are most conveniently edited in spreadsheets (see supplementary material). Initially, all structural sites of tobermorite (Hamid, 1981 ▸; COD 8103550) were described individually within space group *P*1. Ortho-tobermorites were deemed unsuitable because of the implausible coordination of charge-balancing Ca ions located between layers (Richardson, 2014 ▸), but when isolated or turbostratically disordered layers are considered, this issue becomes irrelevant. In order to obtain an isolated layer, the tobermorite structure was reduced to one of its two identical main layers. All developed models are based on this further modified isolated layer. The bridging silicate tetrahedra on one side of the C-(A)-S-H layer were slightly adjusted such that they are symmetric to the other side. Structural sites for charge-balancing Ca ions and respective octahedrally coordinating sites for water or hydroxyl groups were split along **a** and statistically distributed on both sides of the C-(A)-S-H layer (Fig. 1 ▸). Occupancies and vectors for certain atom groups were defined in dummy sites for later adjustment of Ca/Si ratios or atom positions. The maximum bridging silicate tetrahedron occupancy is 0.25 as in tobermorite (Hamid, 1981 ▸), whereas for charge-balancing Ca ions it is 0.5 (Table 1 ▸). Atomic displacement parameters were adopted from the original structure.

Within the supercell, atom positions and interatomic distances are preserved by defining fractional coordinates independent of the lattice constants (Ufer *et al.*, 2004 ▸). For this purpose, variables have to be assigned to the lattice constants and to their original values; the latter are replicated to a dummy site. Multiplication of fractional coordinates with the original lattice constant and division by the current lattice constant cancels out any changes to the latter, as during refinement each fractional coordinate is automatically multiplied by the current lattice constant. Elongation of lattice constants to at least twice their original size creates a partially empty supercell with new *hkl*s simulating even severely asymmetric bands (Ufer *et al.*, 2004 ▸). Consequently, the structure itself accounts for the anisotropic size and macros to account for the anisotropy are no longer necessary. Larger structures within the supercell are created by duplication of structural sites and increment of fractional coordinates; however this may exceed the computational limits of *TOPAS 5.0*. The automatic approximation of *hkl*s using significantly fewer peaks was introduced by *TOPAS 6.0* (Coelho *et al.*, 2016 ▸).

### 3.3. Modification and testing

#### 3.3.1. Single-layer case

**Figure 2:**
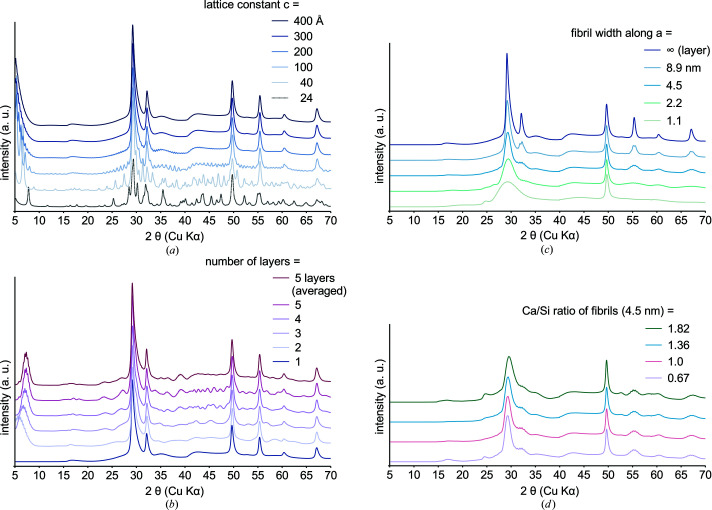
Calculated XRD patterns for C-(A)-S-H featuring isolated layers (*a*), turbostratically disordered layers (11.5 Å layer-to-layer distance) (*b*) and fibrils (*c*), (*d*). The simulations account for a variable divergence slit, an isotropic crystallite size of 30 nm and *b* = 3.675 Å. The Ca/Si ratio was kept at 1.0 (*a*)–(*c*). A non-physical diffraction contribution at low 2θ was removed (*b*)–(*d*).

Isolated C-(A)-S-H layers are simulated by elongation of the supercell along **c**. This can be understood as a two-dimensional crystal structure since the translational symmetry along one dimension is broken. A sufficient number of *hkl*s is produced for *c* = 200 Å [Fig. 2 ▸(*a*)], *ca* ten times the original lattice constant. Complete elimination of oscillations is achieved at *c* > 400 Å. This simplification of turbostratic disorder successfully simulates most asymmetric *hk* bands. A distinct 001 reflection is absent; instead a non-physical 00*l* band appears at <8° 2θ Cu *K*α due to the diffraction contribution from adjacent supercells (Ufer *et al.*, 2004 ▸). In real systems there would be no empty space and nearby atoms cause absorption or diffuse scattering. The redundant 00*l* band can be removed by scaling respective reflections to zero (see *Supplementary material A* ).

#### 3.3.2. Multi-layer case

C-(A)-S-H layer connectivity can occur at low Ca/Si via bridging silicate tetrahedra or at higher Ca/Si via Ca–O octahedra (Grangeon *et al.*, 2016 ▸). Respective turbostratic disorder is implemented by duplication of the C-(A)-S-H layer subjected to stacking vectors. The 001 reflection appears [Fig. 2 ▸(*b*)] and shifts as expected towards 7.4° 2θ Cu *K*α with increasing stack size (Grangeon, Claret, Linard & Chiaberge, 2013 ▸; Grangeon *et al.*, 2016 ▸). The non-physical diffraction contribution at low 2θ is suppressed, but removal of redundant reflections was still applied. Since turbostratic disorder involves random translations and rotations (Ufer *et al.*, 2004 ▸), the disorder obtained from a single set of stacking vectors is insufficient and causes aberrations at >20° 2θ Cu *K*α. Layer rotation may not be implementable, but a combination of *ca* ten multi-layer models with random stacking vectors can average out most aberrations.

#### 3.3.3. Fibrils

Fibrils extending in the **b** direction are simulated by elongation of the lattice constants *a* and *c*. This can be understood as a one-dimensional crystal structure since the translational symmetry along two dimensions is broken. The fibril cross section comprises several original unit cells along **a**, accomplished by duplication of structural sites with an increment of *x*, which relates to the fibril width. The crystallite size parameter can then be interpreted as the fibril length. Small cross sections only produce a broad main band at *ca* 29° 2θ and a 020 reflection at 49.5° 2θ Cu *K*α [Fig. 2 ▸(*c*)]. Increased fibril width increases the translational symmetry along **a** until the XRD patterns resemble the single-layer case. The non-physical diffraction contribution at low 2θ is removed by scaling all *h*0*l* reflections to zero, which would be equivalent to *h* < 1 and *l* < 4 in the original structure (see *Supplementary material A* ).

Further investigation of the fibrillar structure should consider two more hypothetical features of C-(A)-S-H. First, at high Ca/Si ratios charge-balancing Ca–O octahedra have to share O atoms as alternatives are geometrically implausible. In consequence, atoms would be no longer statistically distributed. Instead, chains of charge-balancing Ca–O octahedra are formed along **b** and charge imbalances may occur along **a**. Second, in saturated Ca(OH)_2_ solution the sides of the fibril are more likely to be terminated by Ca–O polyhedra rather than paired silicate tetrahedra. In such a scenario the occupancy of central bridging silicate sites and the mean chain length of silicate tetrahedra could vary without impact on Ca/Si ratios.

### 3.4. Implementation

The developed structure models facilitate the evaluation of experimental results. Layered and fibrillar nanostructures are distinguishable by the shape of the main band at 29.5°, by distinct reflections at 32 and 55° 2θ Cu *K*α, or by the additional presence of a 001 reflection. Considering reported XRD patterns, many laboratory preparations with Ca/Si < 1.5 relate to layers (Black *et al.*, 2006 ▸; Garbev *et al.*, 2008 ▸; Grangeon *et al.*, 2016 ▸; Maddalena *et al.*, 2019 ▸; Roosz *et al.*, 2016 ▸), whereas the XRD patterns of specimens with Ca/Si > 1.5 relate to fibrillar structures (Bergold *et al.*, 2013 ▸; Snellings *et al.*, 2014 ▸). Notably, C-(A)-S-H formed under hydrothermal conditions from reactive silicate sources can be fibrillar even at Ca/Si < 1.0 (Houston *et al.*, 2009 ▸; Kikuma *et al.*, 2011 ▸; Shams *et al.*, 2021 ▸).

Further improvements and specimen-specific modifications will be necessary to account for the whole range of C-(A)-S-H phases. Since even diffuse bands at 42–45° 2θ are precisely simulated, knowledge about the Ca/Si ratio or related occupancies would allow indirect modification of atom groups via refinement of associated vectors. For instance, the positions of charge-balancing Ca^2+^ ions may shift according to Ca/Si with influence on the main band. Although, Grangeon *et al.* (2016 ▸) linked the intensity at 16.1° 2θ to the occupancy of bridging silicate tetrahedra, Ca/Si ratios should be distinguished by complementary methods as their determination via XRD would be ambiguous [Fig. 2 ▸(*d*)].

## 4. Application to autoclaved aerated concrete

### 4.1. Analysis of intermediate C-(A)-S-H

#### 4.1.1. Morphology

**Figure 3:**
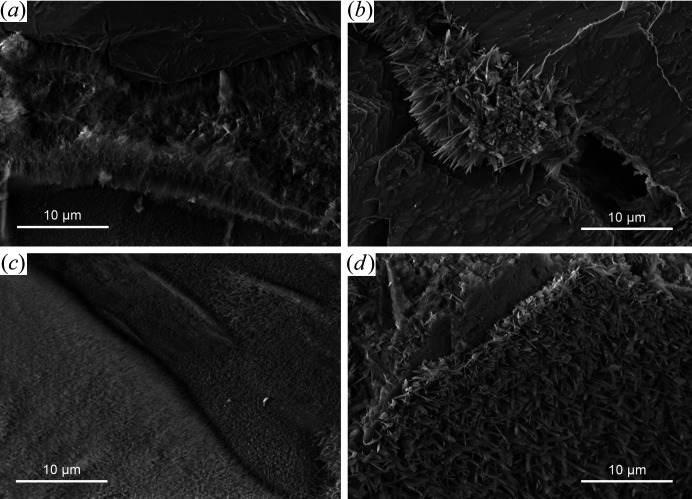
Secondary electron images (10 000×, 5 kV) of quartz grains (*a*), (*b*) and their imprints retained in the fractured porous matrix (*c*), (*d*). Fibrillar C-(A)-S-H (*a*), (*c*) formed around quartz grains within 2 h at 457 K (300 min), whereas tobermorite crystallites (*b*), (*d*) formed after prolonged treatment at 466 K.

**Figure 4:**
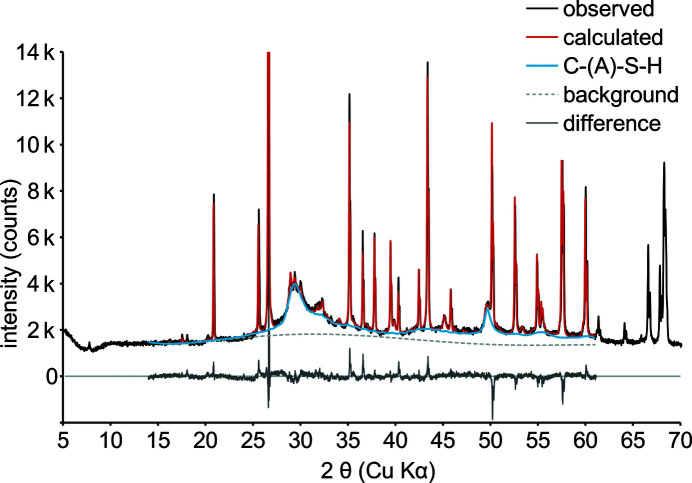
Rietveld refinement of a sample after 1.5 h at 458 K (290 min) with 20 wt% subsequently added corundum.

Secondary electron images of briefly cured AAC reveal C-(A)-S-H fibrils of up to 2 µm in length, often grown towards the surface of dissolving quartz grains [Fig. 3 ▸(*a*)]. Only trace amounts of tobermorite are present at this stage (Fig. 4 ▸). These observations are in agreement with previous studies (Bernstein, 2011 ▸; Mitsuda *et al.*, 1992 ▸) and could be explained by initial restriction of silicate ions to the vicinity of slowly dissolving quartz grains in an otherwise Ca(OH)_2_-saturated hydrothermal solution (Qu *et al.*, 2018 ▸). In contrast, comparable spots in thoroughly cured AAC [Figs. 3 ▸(*b*) and  ▸(*d*)] contain typical lath-like tobermorite (Schreiner *et al.*, 2019 ▸).

#### 4.1.2. Elemental composition

Two samples were analyzed by SEM-EDX: one was prepared with calcium fluoride and did not yield any tobermorite (Table S1), whereas the other did yield small amounts of tobermorite (Table 2 ▸). The obtained Ca/Si and Al/Si ratios are similar to results reported by Mitsuda *et al.* (1992 ▸). The hydrogen and water contents were calculated from oxygen that was not attributable to other quantified oxides. Since this calculation neglects unquantified carbonate, the H_2_O/Si ratios are slightly higher than for other mildly dried samples (Richardson, 2014 ▸). As the hydrothermal reaction proceeds and the quantity of tobermorite increases, the study of C-(A)-S-H by SEM-EDX is hindered and the elemental composition would have to be determined at higher magnifications by TEM-EDX (Mitsuda *et al.*, 1992 ▸; Richardson, 2014 ▸).

#### 4.1.3. Refinement

The observed XRD patterns were best fitted using the 4.5 nm-width fibrillar model (Fig. 4 ▸). The small reflection at 7.9° 2θ was attributable to traces of tobermorite, and the refinement range was reduced to 14–62° 2θ. Furthermore, reduction of the supercell lattice constants to *a* = 200 Å and *c* = 100 Å was necessary to comply with computational limitations of *TOPAS 5.0*. Prior to refinement, the Ca/Si ratio was set to 1.3 according to derived parameters (Table 1 ▸). The crystallite size was reduced to 20 nm in order to match the shape of the 020 reflection at 49.5° 2θ. Stepwise refinement, no matter in which order, led to a main-layer compression of 0.5 Å, an *x* adjustment for charge-balancing Ca^2+^ ions and a *z* adjustment for its coordinating water. In tobermorite (Hamid, 1981 ▸) just half of the main-layer Ca–O polyhedra contain a planar square of O atoms; the other half exhibit a slightly pyramidal geometry. By reducing the fractional coordinate *z* of the latter, the Ca–O polyhedra can be made to share a uniform geometry.

#### 4.1.4. Phase quantification

**Figure 5:**
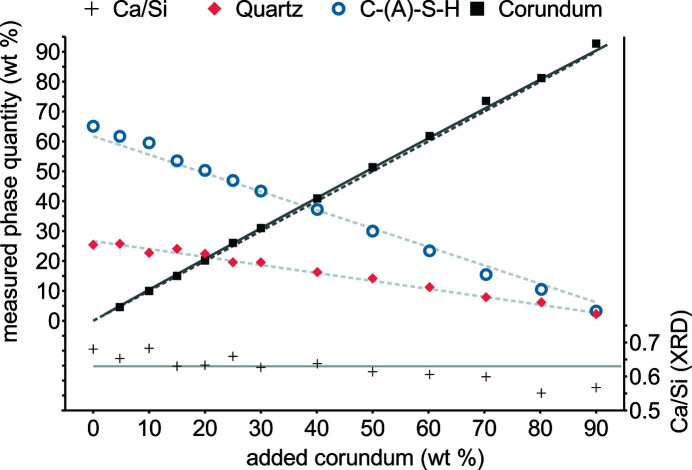
Quantitative XRD of a sample after 1.5 h at *ca* 458 K (290 min) with various amounts of subsequently added corundum. The solid line accounts for 4 wt% amorphous material (*e.g.* adsorbed water). Specimen Ca/Si ratios were calculated on the basis of a Ca/Si ratio of 1.3 for C-(A)-S-H.

The direct quantification of C-(A)-S-H was assessed by the internal standard method (Fig. 5 ▸). Besides quartz and C-(A)-S-H the original sample contains traces of katoite, calcite, larnite, portlandite and tobermorite (Table S2). Here, fifth-order Chebyshev polynomials were chosen; lower orders would slightly exaggerate the C-(A)-S-H quantities and specimen Ca/Si ratios. For quartz, no bias in relation to corundum addition is observed and variations seem to be random, but for C-(A)-S-H there is a small bias. At low corundum additions the trend suggests less then quantified, whereas at high additions the trend suggests more. In conclusion, the direct quantification of C-(A)-S-H via Rietveld refinement yields plausible results, but compared with highly crystalline phases a higher detection limit is expected and interference with background polynomials may occur.

### 4.2. Analysis of hydrothermal reactions

#### 4.2.1. Formation of C-(A)-S-H and tobermorite

**Figure 6:**
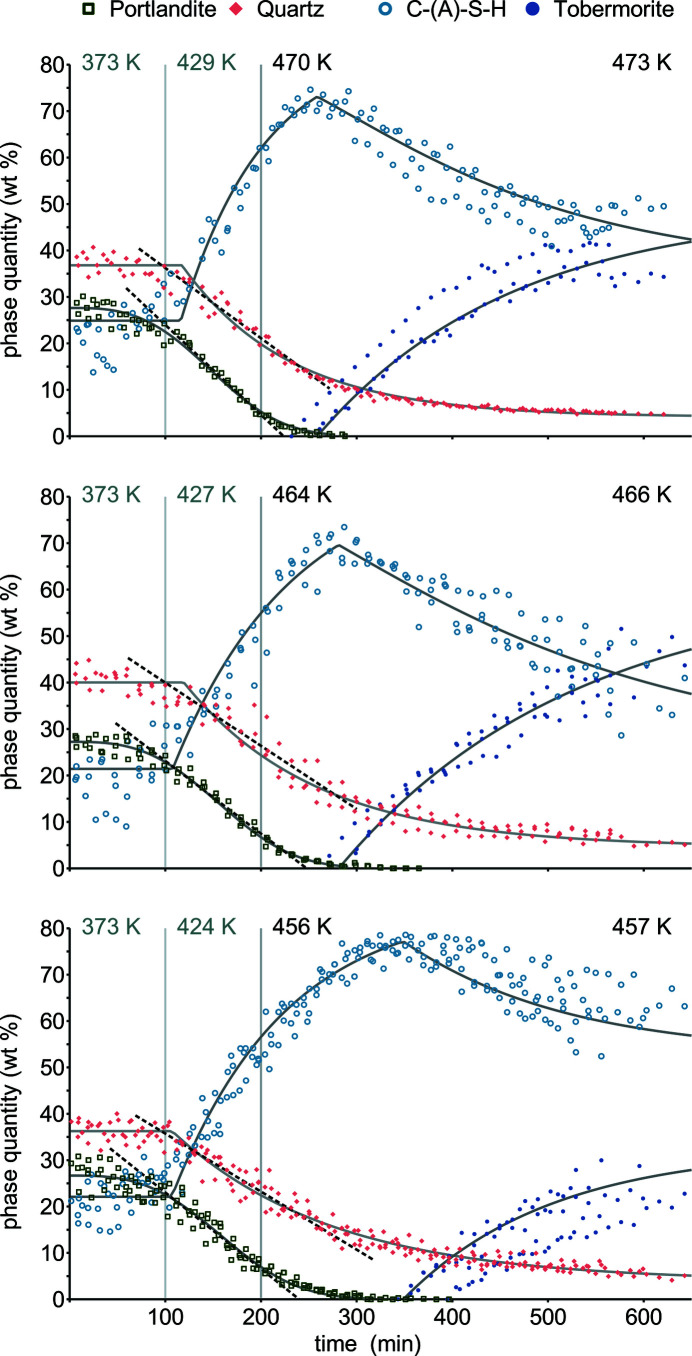
Time-resolved phase quantities combining five *in situ* XRD experiments at 457 K, three at 466 K and three at 473 K. Reaction kinetics are empirically modeled (solid lines) or assumed constant within certain intervals (dashed lines).

**Figure 7:**
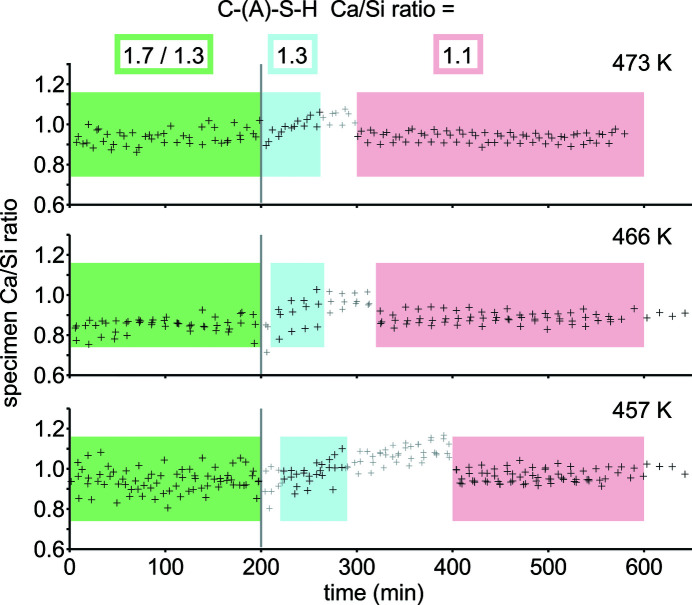
Specimen Ca/Si ratios of *in situ* XRD experiments. The calculations assume Ca/Si = 1.7 for C-(A)-S-H from Portland cement hydration and Ca/Si = 1.3 for additionally formed C-(A)-S-H (100–200 min), then Ca/Si = 1.3 alongside near-complete portlandite consumption (200–300 min), and eventually Ca/Si = 1.1 (300–600 min).

As indicated by the results of *in situ* XRD (Fig. 6 ▸), the hydrothermal reaction of quartz, portlandite and Portland cement can be roughly divided into two stages. The first stage yields C-(A)-S-H and here starts around 425 K (100 min) (Table 3 ▸). This C-(A)-S-H and that from Portland cement hydration were fitted using the refined fibrillar structure model. The Ca/Si ratios of the structure model have a negligible effect on quantitative results and will be discussed later on. For portlandite dissolution, an exponential equation was chosen (Shaw *et al.*, 2000 ▸), which adequately accounts for the temporarily constant reaction rate (100–200 min) and the slowed dissolution of larger remnant crystallites. The dissolution of moderately reactive quartz can be considered as the rate-limiting partial reaction (Matsui *et al.*, 2011 ▸). The second stage is marked by tobermorite formation and conversion of C-(A)-S-H; in the corresponding kinetic models, the increase in tobermorite is balanced by a decrease in C-(A)-S-H content. At 466 and 473 K (*ca* 260 min), tobermorite formation started soon after complete portlandite consumption, whereas at 457 K it was significantly delayed. Since the determined reaction rates do not reflect this temperature correlation, the onset of tobermorite formation is seen as the most useful parameter for the comparison of experiments. The surface sensitivity of *in situ* XRD measurements using Bragg–Brentano geometry can be problematic (Mesecke *et al.*, 2020 ▸). The phase quantities before hydrothermal reaction were calculated from the raw materials, assuming cement hydration yielded 25 wt% portlandite (Taylor, 1997 ▸), and afterwards control measurements were performed on homogenized samples (Table 4 ▸). From the resulting data it can be concluded that the surface composition differed from the bulk, and *in situ* measurements observe *ca* 10 wt% more C-(A)-S-H and 10 wt% less quartz. Hence the specimen Ca/Si ratios (Fig. 7 ▸) are higher than expected.

#### 4.2.2. Evolution of Ca/Si ratios

The calculated specimen Ca/Si ratios reflect the varying Ca/Si ratios of C-(A)-S-H (Fig. 7 ▸). Assumption of constant Ca/Si ratios would imply an implausible increase of specimen Ca/Si ratios (300–400 min). Initially, Ca/Si = 1.7 can be expected for C-(A)-S-H from Portland cement hydration (Taylor, 1997 ▸). For C-(A)-S-H formed during the first stage, Ca/Si ratios of 1.41, 1.23 and 1.37 are indicated by the ratio of constant reaction rates for portlandite and quartz dissolution (100–200 min) divided by the respective molar masses. When the Ca(OH)_2_ concentration and alkalinity in hydrothermal solution decreases (200–300 min) (Mitsuda, 1982 ▸), the Ca/Si ratios of the initial C-(A)-S-H are also expected to decrease (Baston *et al.*, 2012 ▸; Lothenbach & Nonat, 2015 ▸). Intermediate Ca/Si ratios of 1.31–1.35 were determined by SEM-EDX. An eventual Ca/Si ratio of 1.1, as observed by Mitsuda *et al.* (1992 ▸) using TEM-EDX, would render the specimen Ca/Si ratios of subsequent XRD measurements consistent with expectations (Table 4 ▸).

#### 4.2.3. Other phases

Additionally, *in situ* experiments observed katoite (Fig. S2), larnite (Fig. S3) and calcite (Fig. S4). Although calcium sulfates and ellestadite-(OH) are common for AAC, none were observed in these sulfate-reduced samples. Katoite appeared at 373 K (0 min) alongside the decay of calcium aluminium oxide hydrates with reflections at *ca* 11° 2θ Cu *K*α (Jensen *et al.*, 2005 ▸). Katoite dissolved slowly during both stages of hydrothermal reaction, and aluminate was presumably incorporated into the formed C-(A)-S-H and tobermorite. Unhydrated larnite from the cement, initially present at *ca* 2 wt%, dissolved alongside the near-complete portlandite consumption. The calcite quantities increased due to surface carbonatization from *ca* 2 wt% initially to occasionally 5 wt%. Fluorite (Fig. S5) is not as inert as expected and cannot serve as an internal standard. SEM-EDX confirms calcium fluoride uptake by C-(A)-S-H (Table S1) and the decrease corresponding to near-complete portlandite consumption was also reflected by XRD measurements of subsequently homogenized samples (Table 4 ▸).

### 4.3. Analysis of industrially produced AAC

**Figure 8:**
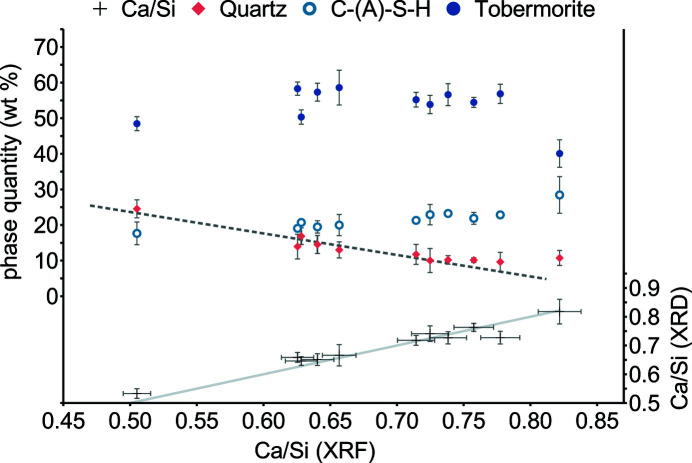
Quantitative XRD of industrially produced AAC with raw densities of *ca* 350 kg m^−3^. Specimen Ca/Si ratios were determined via XRF or calculated on the basis of a Ca/Si ratio of 0.9 for C-(A)-S-H. Error bars represent the 2σ repeatability interval.

The feasibility of direct C-(A)-S-H quantification is further demonstrated on 11 AAC products (Fig. 8 ▸) (Table S3). XRD patterns were best fitted using the 8.9 nm-width fibrillar model, which is attributable to a slightly more lamellar morphology. There is a trend to more C-(A)-S-H at higher specimen Ca/Si ratios, whereas quartz quantities decrease linearly up to Ca/Si = 0.75 (Fig. 8 ▸, dashed line). At the time of analysis, the products were 1–6 months old and already contained some vaterite. Since C-(A)-S-H is susceptible to carbonatization, its Ca/Si ratio probably decreased by a loss of charge-balancing Ca without silicate uptake. Here the assumption of Ca/Si = 0.9 provides consistent results. Specimen Ca/Si ratios diverging from the trendline may display different degrees of carbonatization.

## 5. Conclusions

Atomistic structure models of C-(A)-S-H combined with a supercell approach using *TOPAS* can be used to satisfactorily refine various XRD patterns by accounting for nanostructural features like isolated layers, turbostratically disordered layers and fibrils. Such supercells are obtained by an elongation of lattice constants and disruption of translational symmetry. Layers require the loss of one dimension and fibrils require the loss of two. Asymmetric *hk* bands and even diffuse bands are precisely simulated; large-scale models accounting for numerous defects are not necessary. However, a prerequisite for Rietveld refinement is a complementary compositional analysis or a justifiable assumption for the occupancies of bridging silica tetrahedra, charge-balancing Ca ions and respective Ca/Si ratios. The hydrothermal reaction of portlandite with moderately reactive quartz initially produces fibrillar C-(A)-S-H at Ca/Si ratios of 1.3–1.4, which decrease as the reaction proceeds or as the final product carbonizes. It is shown that the direct quantification of C-(A)-S-H via structure models yields plausible results and has fewer demands in terms of sample preparation and measurement conditions than internal or external standard methods. The structure models are utilizable for hydrothermally cured building materials and presumably also Portland cement concrete.

## Supplementary Material

: https://doi.org/10.5281/zenodo.5861604

Crystal structure: contains datablock(s) znvo, I. DOI: 10.1107/S1600576721012668/vb5025sup1.cif

Rietveld powder data: contains datablock(s) C-S-H_20_corundum. DOI: 10.1107/S1600576721012668/vb5025Isup2.rtv

CIF data for un-refined single layer model with Ca/Si 1.0. DOI: 10.1107/S1600576721012668/vb5025sup3.txt

CIF data for refined fibrillar model with Ca/Si 0.9. DOI: 10.1107/S1600576721012668/vb5025sup4.txt

CIF data for refined fibrillar model with Ca/Si 1.1. DOI: 10.1107/S1600576721012668/vb5025sup5.txt

CIF data for refined fibrillar model with Ca/Si 1.3. DOI: 10.1107/S1600576721012668/vb5025sup6.txt

STR file for un-refined single layer model with Ca/Si 1.0. DOI: 10.1107/S1600576721012668/vb5025sup7.txt

Development and documentation of the structure model which is suitable for further modification. DOI: 10.1107/S1600576721012668/vb5025sup8.xlsx

STR file for un-refined five-layer model with Ca/Si 1.0. DOI: 10.1107/S1600576721012668/vb5025sup9.txt

PRO file for the refinement of XRD data related to Fig.8. DOI: 10.1107/S1600576721012668/vb5025sup10.txt

PRO file for the refinement of XRD data related to Fig.6. DOI: 10.1107/S1600576721012668/vb5025sup11.txt

PRO file for the refinement of XRD data related to Fig.4 and Fig.5. DOI: 10.1107/S1600576721012668/vb5025sup12.txt

Supplemental material A (supplementary tables and figures). DOI: 10.1107/S1600576721012668/vb5025sup13.pdf

STR file for refined fibrillar model with Ca/Si 0.9. DOI: 10.1107/S1600576721012668/vb5025sup14.txt

STR file for refined fibrillar model with Ca/Si 1.1. DOI: 10.1107/S1600576721012668/vb5025sup15.txt

STR file for refined fibrillar model with Ca/Si 1.3. DOI: 10.1107/S1600576721012668/vb5025sup16.txt

CCDC reference: 2124994

## Figures and Tables

**Table 1:** Idealized compositional parameters for isolated C-(A)-S-H layers summarized from previous studies (Gartner *et al.*, 2017 ▸; Richardson, 2014 ▸) The occupancy parameter for the respective structural site is given in brackets.

			Charge balancing		
Ca/Si	H_2_O/Si	Bridging silicate at.%	H^+^ at.%	Calcium at.%	CaOH^+^ at.%
0.67	0.3	100 (0.25)	100	0 (0.0)	0
0.75	0.5	86 (0.214)	86	7 (0.035)	0
1.0	1.0	50 (0.125)	50	25 (0.125)	0
1.273	1.5	20 (0.05)	20	40 (0.2)	0
1.364	1.7	20 (0.05)	0	50 (0.25)	0
1.5	2.0	20 (0.05)	0	65 (0.325)	30
1.7	2.4	20 (0.05)	0	87 (0.435)	85
1.82	2.6	20 (0.05)	0	100 (0.5)	100

**Table 2:** Elemental composition of C-(A)-S-H analyzed by SEM-EDX for a sample after 1.5 h at 458 K (290 min) Hydrogen quantification (*) assumes excess oxygen to be present as water. The results of 17 spectra were averaged.

Atom	Atom%	Wt%	Oxide	Wt%	σ
*H		2.58	H_2_0	23.20	5.72
O	70.62	51.14			3.11
Na	0.06	0.07	Na_2_0	0.09	0.11
Mg	0.11	0.12	MgO	0.20	0.08
Al	0.36	0.44	Al_2_O_3_	0.84	0.31
Si	12.35	15.66	SiO_2_	33.50	2.47
S	0.13	0.19	SO_3_	0.47	0.13
K	0.05	0.09	K_2_O	0.11	0.12
Ca	16.03	28.99	CaO	40.57	2.96
Fe	0.28	0.72	Fe_2_O_3_	1.02	0.23
Ca/Si	1.31				0.07
Ca/(Al + Si)	1.27				0.06
Al/Si	0.03				0.01
Al/(Al + Si)	0.03				0.01
H_2_O/Si	2.36				0.73

**Table:** Portlandite: *w*(*t*) = *w*_0_exp(−*kt* ^3^).

	457 K	466 K	473 K
*w*_0_ (wt%)	27	27	28
*k* (s^−3^)	7.8 × 10^−13^	8.1 × 10^−13^	9.5 × 10^−13^

**Table:** Quartz: *w*(*t*) = (*w*_0_ − *w*_\infty_)exp[−*k*(*t* − *t*_0_)] + *w*_\infty_.

	457 K	466 K	473 K
*w*_0_ (wt%)	36	40	37
*w*_\infty_ (wt%)	4	5	4
*k* (s^−1^)	1.0 × 10^−4^	1.2 × 10^−4^	1.5 × 10^−4^
*t*_0_ (min)	107	119	117

**Table:** C-(A)-S-H: *w*(*t*) = (*w*_\infty_ − *w*_0_) {1 − exp[−*k*(*t* − *t*_0_)]} + *w*_0_.

	457 K	466 K	473 K
*w*_0_ (wt%)	22	21	25
*w*_\infty_ (wt%)	85	86	84
*k* (s^−1^)	1.4 × 10^−4^	1.3 × 10^−4^	1.9 × 10^−4^
*t*_0_ (min)	107	108	116

**Table:** Tobermorite: *w*(*t*) = *w*_\infty_ {1 − exp[−*k*(*t* − *t*_0_)]}.

	457 K	466 K	473 K
*w*_\infty_ (wt%)	32	61	49
*k* (s^−1^)	1.1 × 10^−4^	0.7 × 10^−4^	0.8 × 10^−4^
*t*_0_ (min)	349	281	259

**Table 4:** Phase quantities (wt%) before *in situ* experiments as calculated from the raw materials and afterwards as determined by XRD measurements of homogenized samples Specimen Ca/Si ratios were calculated on the basis of a Ca/Si ratio of 1.1 for C-(A)-S-H.

	Before	457 K	466 K	473 K
Portlandite	27.6	–	–	–
Larnite	2.1	–	–	–
Quartz	48.3	18.9	17.9	18.1
C-(A)-S-H	10.0	48.2	40.4	39.7
Tobermorite	–	24.0	32.7	32.9
Katoite	5.4	4.3	3.6	3.8
Calcite	2.1	2.0	3.1	3.0
Fluorite	4.7	2.6	2.4	2.5
Ca/Si	0.69	0.70	0.70	0.70
