# Supplementary material for: Structure modeling and quantitative X-ray diffraction of C-(A)-S-H
Source: J Appl Crystallogr. 2022 Feb 1;55(Pt 1):133–43. doi: 10.1107/S1600576721012668 (PMC8805164; doi:10.1107/S1600576721012668)
Supplement: Supplementary file 13 [file j-55-00133-sup13.pdf]

Supplemental material A:
Structure modelling and quantitative X-ray diffraction of C-(A)-S-H

Karsten Mesecke,[a,b]* Laurence N. Warr[b] and Winfried Malorny[a]

[a] *Hochschule Wismar, Philipp-Müller-Straße 14, 23966 Wismar Germany, and* [b] *University of Greifswald, Friedrich-Ludwig-Jahn-Straße 17A, 17489 Greifswald Germany.*

E-mail: karsten.mesecke@stud.uni-greifswald.de

Removal of redundant $00l$ reflections produced by the single layer model:

```
scale_pks = If(And((K==0),(H==0),Abs(L)<(c_8103550/c0_8103550*3.8)),0,1);
```

Removal of redundant $00l$ reflections produced by the two-layer model:

```
scale_pks = If(And((K==0),(H==0),Abs(L)<(c_8103550/c0_8103550*3.8)),0,1);
```

Removal of redundant $00l$ reflections produced by the three-layer model:

```
scale_pks = If(And((K==0),(H==0),Abs(L)<(c_8103550/c0_8103550*3.8)),0,1);
```

Removal of redundant $00l$ reflections produced by the four-layer model:

```
scale_pks = If(And((K==0),(H==0),Abs(L)<(c_8103550/c0_8103550*3.8)),0,1);
```

Removal of redundant $00l$ reflections produced by the five-layer model:

```
scale_pks = If(And((K==0),(H==0),Abs(L)<(c_8103550/c0_8103550*3.8)),0,1);
```

Removal of redundant $h0l$ reflections produced by the fibrillar model:

```
scale_pks = If(And((K==0),Abs(H)<(a_8103550/a0_8103550),Abs(L)<(c_8103550/c0_8103550*3.8)),0,1);
```

Table 1. *Elemental composition of C-(A)-S-H analyzed by SEM-EDX for a sample with calcium fluoride after 2 h at 457 K (300 min). Hydrogen quantification (*) assumes excess oxygen to be present as water. Results of 9 spectra were averaged.*

	atom %	wt%	oxide	wt%	σ
*H		2.40	H_20	21.63	2.09
O	68.80	49.51			1.15
F	1.20	1.02	CaF_2	2.10	0.40
Na	0.13	0.13	Na_20	0.18	0.04
Mg	0.16	0.18	MgO	0.30	0.20
Al	0.50	0.60	Al_2O_3	1.14	0.50
Si	12.24	15.45	SiO_2	33.06	1.20
S	0.11	0.16	SO_3	0.40	0.14
K	0.10	0.18	K_2O	0.22	0.07
Ca	16.50	29.73	CaO	40.09	1.00
Fe	0.25	0.62	Fe_2O_3	0.89	0.18
Ca/Si			1.35		0.05
Ca/(Al+Si)			1.30		0.05
Al/Si			0.04		0.02
Al/(Al+Si)			0.04		0.02
H_20/Si			2.19		0.26

Table 2. *Detailed refinement results for mixtures with corundum (Fig. 5).*

Corundum wt% added	-	4.8	10.0	15.0	20.0	25.0	30.0	40.1	50.0	60.2	70.3	80.2	90.0
Corundum	0.0	4.6	10.0	15.0	20.2	26.1	31.0	41.0	51.4	61.8	73.6	81.2	92.7
Portlandite	0.4	0.3	0.3	0.2	0.2	0.2	0.2	0.2	0.1	0.1	0.1	0.1	0.1
Larnite	0.7	0.6	0.4	0.6	0.5	0.5	0.3	0.3	0.2	0.1	0.0	0.0	0.0
Quartz	25.5	25.8	22.7	24.1	22.4	19.6	19.6	16.3	14.2	11.2	7.9	6.2	2.3
C-(A)-S-H	65.0	61.7	59.5	53.5	50.3	46.9	43.4	37.2	30.0	23.4	15.5	10.5	3.3
Tobermorite	4.5	3.7	3.6	3.4	3.4	3.8	3.1	3.0	2.2	2.1	1.8	1.3	1.2
Katoite	3.4	3.1	3.1	2.8	2.8	2.6	2.3	2.0	1.8	1.3	1.1	0.7	0.4
Calcite	0.6	0.3	0.5	0.3	0.3	0.5	0.2	0.2	0.1	0.0	0.1	0.0	0.0
Ca/Si (XRD)	0.68	0.65	0.68	0.63	0.63	0.66	0.63	0.64	0.61	0.61	0.60	0.55	0.57
R_{wp}	4.4	4.5	4.4	4.9	4.7	4.7	4.7	5.2	5.7	6.0	6.2	6.5	7.1

Table 3. *Detailed refinement results for industrial products each averaged from three measurements (Fig. 8).*

No.	1	2	3	4	5	6	7	8	9	10	11
						wt%					
Quartz	24.5	13.9	16.9	14.5	13.0	11.8	10.0	10.2	10.1	9.6	10.7
C-(A)-S-H	17.6	19.1	20.7	19.5	20.0	21.3	22.9	23.3	21.9	22.9	28.4
Tobermorite	48.5	58.5	50.3	57.3	58.6	55.2	53.9	56.6	54.5	56.8	40.1
Katoite	1.9	1.7	1.6	1.2	1.2	2.6	2.5	1.6	1.9	1.1	3.7
Calcite	3.0	2.7	2.1	1.7	0.8	4.3	2.2	2.1	5.0	1.8	7.3
Vaterite	1.2	1.6	3.1	2.5	2.1	1.9	1.3	2.4	2.7	1.1	1.2
Anhydrite	2.9	2.3	0.8	2.9	3.9	2.5	1.1	3.5	2.3	1.6	0.5
Bassanite	-	-	1.2	-	-	-	0.5	-	-	0.5	0.7
Gypsum	-	-	1.2	-	-	-	1.6	-	-	1.8	2.7
Ellestadite-(OH)	-	-	2.0	-	-	-	3.7	-	1.2	2.5	4.3
Orthoclase	0.2	0.1	0.1	0.1	0.2	0.2	0.2	0.1	0.2	0.1	0.1
Phlogopite	0.2	0.3	0.1	0.3	0.2	0.3	0.1	0.2	0.2	0.1	0.2
Ca/Si (XRF)	0.51	0.63	0.63	0.64	0.66	0.71	0.72	0.74	0.76	0.78	0.82
Ca/Si (XRD)	0.53	0.66	0.65	0.65	0.67	0.72	0.74	0.73	0.76	0.73	0.82
R_{wp}	7.5	6.4	6.3	6.7	6.5	6.3	6.3	5.6	5.6	6.4	6.0
	7.2	6.8	6.4	6.3	7.1	6.6	6.3	6.1	6.0	7.0	6.9
	7.0	7.1	5.7	6.8	6.5	5.8	5.7	6.2	5.8	6.2	5.7

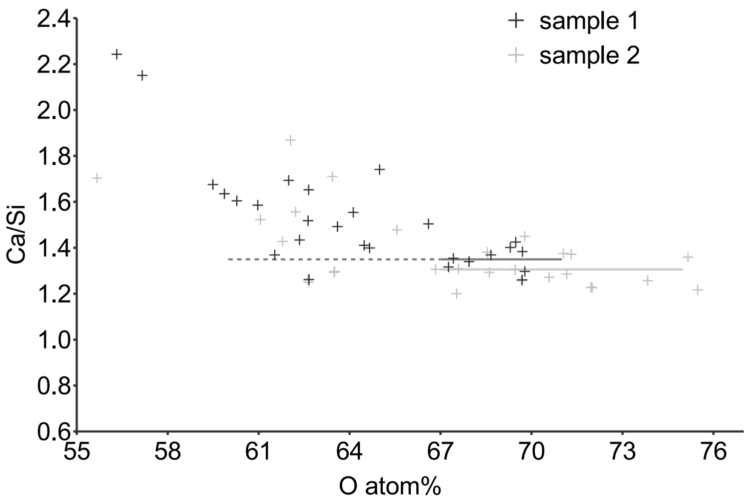

Fig. 1. Correlation of Ca/Si ratios and oxygen atom% as a selection criteria for SEM-EDX evaluation.

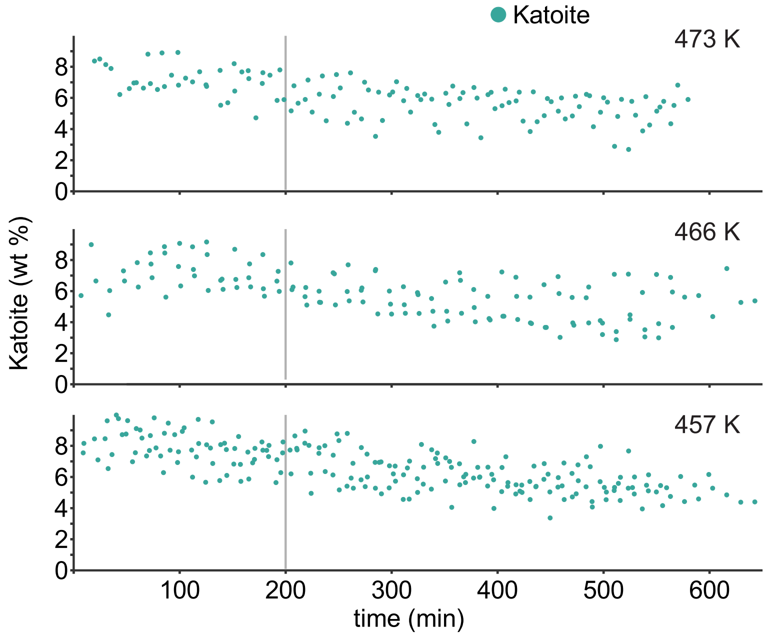

Fig. 2. The dissolution of katoite observed by *in situ* experiments.

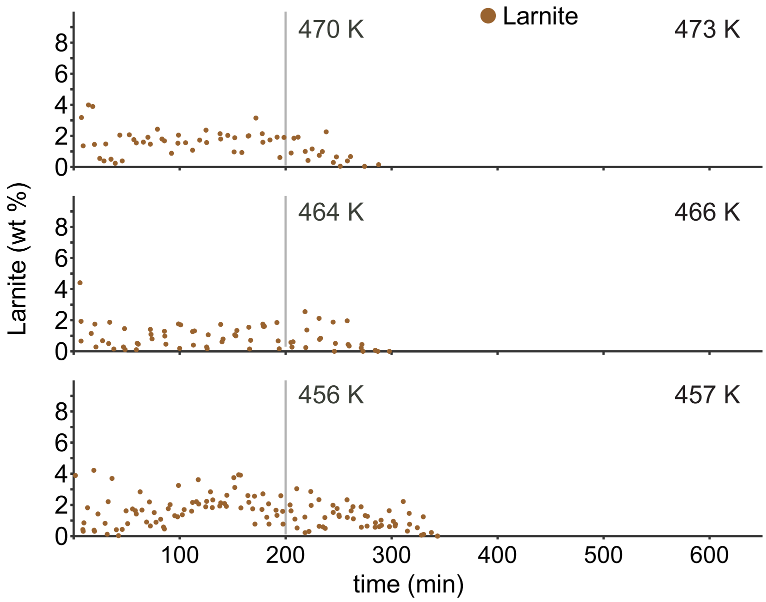

Fig. 3. The dissolution of larnite observed by *in situ* experiments.

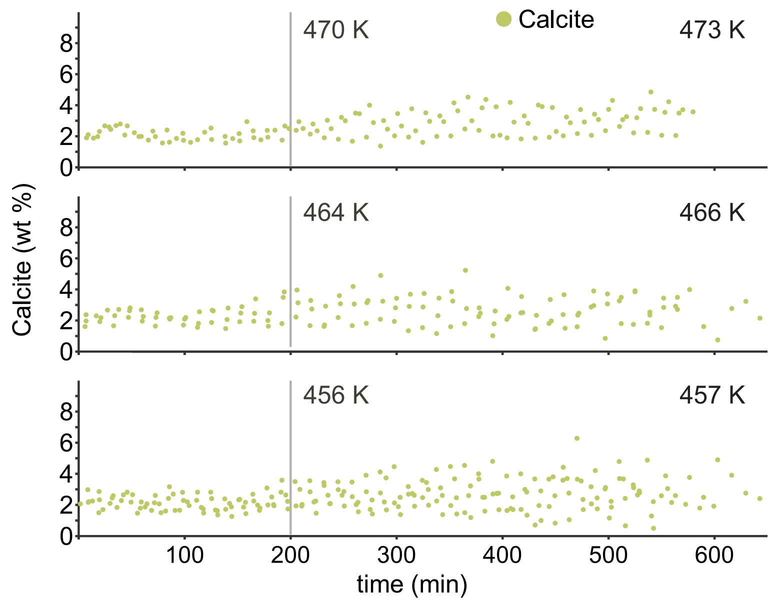

Fig. 4. The quantity of calcite observed by *in situ* experiments.

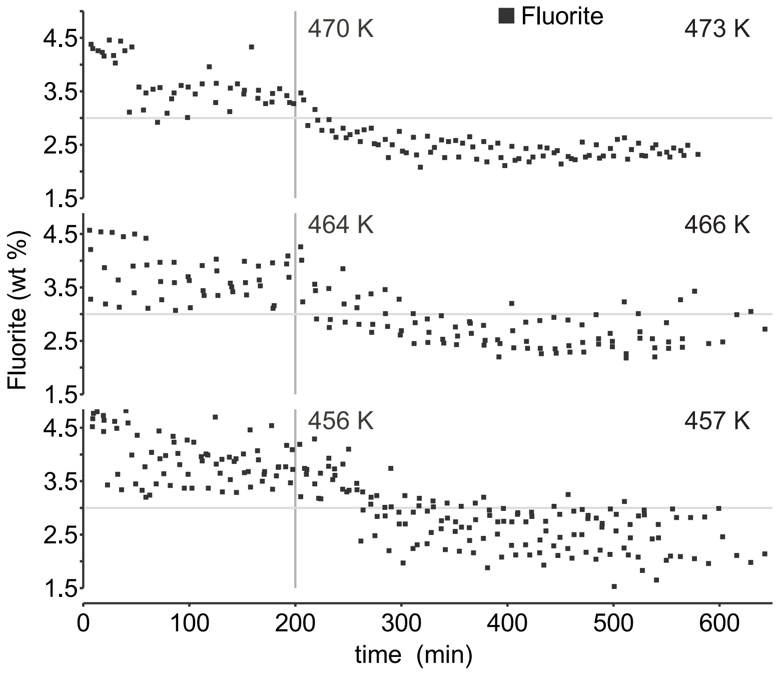

Fig. 5. The dissolution of fluorite observed by *in situ* experiments.
